# Perfusion CT detects alterations in local cerebral flow of glioma related to IDH, MGMT and TERT status

**DOI:** 10.1186/s12883-021-02490-4

**Published:** 2021-11-24

**Authors:** Ke Wang, Yeming Li, Haiyang Cheng, Shenjie Li, Wei Xiang, Yang Ming, Ligang Chen, Jie Zhou

**Affiliations:** 1 Department of Neurosurgery, Affliated Hospital of Southwest Medical University, Luzhou, China; 2 Department of Neurosurgery, The General Hospital of Western Theater Command PLA, Chengdu, China; 3 Neurosurgery Clinical Medical Research Center of Sichuan Province, Lu Zhou, China; 4 Neurological Diseases and Brain Function Laboratory, Luzhou, China; 5 Southwest Medical University, Luzhou, China

**Keywords:** Glioma, Molecular pathology, Perfusion CT

## Abstract

**Background:**

The aim of this study was to investigate the relationship between tumor biology and values of cerebral blood volume (CBV), cerebral blood flow (CBF), mean transit time (MTT), time to peak (TTP), permeability surface (PS) of tumor in patients with glioma.

**Methods:**

Forty-six patients with glioma were involved in the study. Histopathologic and molecular pathology diagnoses were obtained by tumor resection, and all patients accepted perfusion computed tomography (PCT) before operation. Regions of interests were placed manually at tumor and contralateral normal-appearing thalamus. The parameters of tumor were divided by those of contralateral normal-appearing thalamus to normalize at tumor (relative [r] CBV, rCBF, rMTT, rTTP, rPS). The relationships of the parameters, world health organization (WHO) grade, molecular pathological findings were analysed.

**Results:**

The rCBV, rMTT and rPS of patients are positively related to the pathological classification (*P* < 0.05). The values of rCBV and rPS in IDH mutated patients were lower than those IDH wild-type. The values of rCBF in patients with MGMT methylation were lower than those MGMT unmethylation (*P* < 0.05). The MVD of TERT wild-type group was lower than TERT mutated group (*P* < 0.05). The values of rCBV were significant difference in the four molecular groups divided by the combined IDH/TERT classification (*P* < 0.05). The progression free survival (PFS) and overall survival (OS) were significant difference in the four molecular groups divided by the combined IDH/TERT classification (*P* < 0.05).

**Conclusions:**

Our study introduces and supports the changes of glioma flow perfusion may be closely related to its biological characteristics.

**Supplementary Information:**

The online version contains supplementary material available at 10.1186/s12883-021-02490-4.

## Background

Glioma constitutes a systemic disease of the brain with tumor cells spreading far beyond the macroscopically visible lesion and form networks throughout the whole brain [1]. The WHO grading system classified it as I-IV grades, with worst prognosis in grade IV gliomas. However, with the discovery and research of glioma gene targets in recent years, the subtypes of glioma are re-stratified. On the basis of molecular pathology diagnosis results, previous researches have suggested that the tumor markers such as IDH mutation status [2], MGMT promoter methylation [3] and TERT promoter mutation status [4] and so on are independently or interactively associated with the disease free survival and the overall survival in glioma patients, even affecting the operation and concurrent chemo-radiotherapy of glioma patients. Studies found that IDH mutations are considered to be an early event in glioma development, and IDH mutations appear to lead to cell state permissive of transformation, possibly leading to blocked cell differentiation and promoting cell proliferation [5], which is closely related to prognosis. MGMT may lead to drug resistance of tumor cells to alkylating agents by allowing DNA repair to glioma cells. MGMT promoter methylation may reduce MGMT activity, thus inhibiting the repair of DNA damages after radiation and chemotherapy [6]. TERT encodes the catalytic subunit of telomerase, and telomere length in normal cells is usually shortened after each cell cycle, leading to cell senescence and apoptosis. Mutations in the TERT promoter can improve gene transcription, leading to increased TERT mRNA levels [7], which predict a poor prognosis in glioma patients [8]. These viewpoints provide us with certain clues to the role of each gene in the development and development of glioma. However, the mechanism of these genes’ influences on glioma is still not completely clear.

Glioma is characterized by abnormal vasculature with angiogenesis, which is a typical tumor hallmark participating in multiple biological behaviors such as tumor progression, invasiveness, and therapy resistance [9]. In recent years, with the development of non-invasive brain perfusion imaging technology, previous studies have reported that relevant parameters can obtain the hemodynamic information of glioma, summarize its microvascular environment [10], and characterize gliomas of different grades [11, 12]. Early PCT studies of glioma often focused on the differential diagnosis and tumor pathological grade [13–15]. However, there are few studies on the relationship between the status of IDH, MGMT, TERT and perfusion indicators in glioma patients. Our study aims to explore the correlation between tumor grade, the status of IDH, MGMT, TERT and tumor perfusion indicators in glioma patients. Furthermore, our study firstly detects the differences of perfusion parameters, PFS, OS in the utility of molecular classification based on the IDH and TERT statuses in newly diagnosed WHO grade II- IV diffuse gliomas and in the utility of molecular classification based on the MGMT and TERT statuses in newly diagnosed glioblastoma (GBM).

## Materials and methods

### Subjects

We reviewed the records of 46 consecutive patients that underwent preoperative PCT for newly diagnosed glioma from January 2018 to November 2018 in the Department of Neurosurgery, the Affiliated Hospital of Southwest Medical University. The inclusion criteria were: (1) had complete clinical data, included preoperative PCT. (2) pathologically confirmed glioma. (3) had glioma histopathologic diagnosises and molecular pathologic diagnosises. (4) patients volunteered to participate in the research. (5) right-handedness. The exclusion criteria were: (1) recurrent glioma patients. (2) previous brain biopsy or surgery. (3) previous radiation or chemotherapy. (4) had other cerebral system diseases. At last, forty-six cases were enrolled, including 22 males and 24 females, the range of age from15 ~ 83 (48.39 ± 13.54) years old. Postoperative pathological diagnosis was gliomas, including WHOI grade 1 case, II grade 15 cases, III grade 11 cases, IV grade 19 cases (Table 1).

**Table 1:** Baseline characteristics of the patients with glioma

Characteristics	No. (46)	Proportion (%)
**Ages** (ys), (Mean ± SD)	48.39 ± 13.54	
**Gender** (male/female)	22/24	47.8/52.2
**Grade**		
WHO I	1	2.2
WHO II	15	32.6
WHO III	11	23.9
WHO IV	19	41.3
**Tumor Histology**		
Pilocytic Astrocytoma	1	2.2
Oligodendroglioma	6	13.0
Diffuse Astrocytoma	8	17.4
Pleomorphic Xantho Astrocytoma	1	2.2
Anaplastic Oligodendroglioma	7	15.2
Anaplastic Astrocytoma	4	8.7
Glioblastoma	19	41.3
**Location**		
On the tentorium cerebelli	44	95.7
Under the tentorium cerebelli	2	4.3
**IDH**		
Mutation	21	45.7
Wild	25	54.3
**TERT**		
Mutation	29	63
Wild	17	37
**MGMT**		
Methylation	33	71.1
Unmethylation	13	28.3

### Grouping methods

According to the results of latest studies of clMPACT-NOW and studies have been carried out to classify glioma subtypes based on the combined IDH/TERT status in patients with II-IV diffuse glioma [16–18], forty-four patients in our study were divided into four groups. Group A was grade II-IV diffuse glioma with IDH wild-type and TERT mutation, Group B was grade II-IV diffuse glioma with IDH wild-type and TERT wild-type, Group C was grade II-IV diffuse glioma with IDH mutation and TERT mutation, Group D was grade II-IV diffuse glioma with IDH mutation and TERT wild-type. Moreover, based on the combined MGMT/TERT status in patients with GBM, nineteen patients were divided into four groups. Group A was GBM with MGMT methylation and TERT wild-type, Group B was GBM with MGMT methylation and TERT mutation, Group C was GBM with MGMT un-methylation and TERT wild-type, Group D was GBM with MGMT un-methylation and TERT mutation.

### Regions of interests (ROIs) selection

The ROIs of glioma were placed manually at multidimensional parenchymal areas of tumors [11, 14, 15, 19], which the CBV, CBF, MTT, TTP, PS of tumor parenchyma were measured at different levels and multiple points, and the final perfusion parameters of tumors were averaged. Meanwhile, the perfusion parameters of the contralateral normal-appearing thalamus were used as normal control. The averaged parameters of tumor were divided by those of contralateral normal-appearing thalamus to normalize at tumor (relative [r] CBV, rCBF, rMTT, rTTP, rPS). These ROIs were not excluded, which were closed to the tumor necrosis, tumor cyst, edema, and difficult to distinguish the anatomical structures.

### PCT protocol

Firstly, a noncontrast-enhanced CT scan was processed in all patients by Philips Briliancei 256-slice spiral CT scanner after patients had iodine anaphylactic test and the result was negative. Sceondly, the contrast agent (Iobitridol, 350 mgI/ml) was given rapidly (6 ml/s) through an elbow intravenous bolus injection with an automatic injector (2 mL/kg). Thirdly, normal saline (30 ml) was injected with the same speed. After 5 s delay, scanning was performed at the parameters of 80 kVs, 100 mAs, 0.4 s/cycle, 4.1 s interval, 13 cycles totally, 5 mm slice thickness, 512*512 matrix, 54.4 s contrast agent tracking time and 12.8 cm coverage. At last, the reorganized dynamic images transmitted to the workstation, which were processed in Philips Extended Brilliant Workstation using CT brain perfusion software.

### PCT data processing and analysis

Two experienced radiologists were responsible for measuring perfusion parameters in Philips Extended Brilliant Workstation using CT brain perfusion software, who were blinded to the clinical results of patients. If two radiologists had conflicted opinions, a third radiologist was involved in the evaluation. The input artery was the ascending petrous segment of the internal carotid artery, and the output vein was the superior sagittal sinus. Combined with preoperative magnetic resonance imaging of patients, radiologist and neurosurgeon manually draw ROIs (21mm^2^), avoiding the necrotic or cystic parts of the tumor and cortical vessels, to generate the time density curve, the false-color images and perfusion parameters of ROIs, including CBF, CBV, MTT, TTP, and PS. The ROIs parameter values were corrected by the value of hematocrit.

### Microvessel density (MVD) data processing and analyses

a. Pathological section preparation: Tumor paraffin-embedded tissue blocks were taken, and 4 sections were made successively, with a thickness of 4 μm. b. Reagents and immunohistochemical staining: Antibodies and detection systems: The antibodies and detection system used in this study were all products of Beijing Zhongshan Jinqiao Biotechnology Co., Ltd., and were CD34 monoclonal antibody, EnVision (Polymer) two-step PV-9000 reagent, 0.01 mol/L phosphate buffer (PBS, pH 7.2 ~ 7.4), 0.01 mol/L citrate buffer (pH 6.0). Immunohistochemical staining was performed PV6000 system. Immunohistochemical staining of CD34 was performed. c. MVD counting: Weidner method was used to determine the positive results. First, the area with the highest vascular density of the tumor was found at low magnification (× 100), and then the number of microvessels in the five areas with the highest vascular density was counted at high magnification (× 400), and the mean value was taken to represent MVD.

### Statistical analyses

SPSS 22.0 statistical software was used for statistical analysis. The number of count data cases (percentage) was expressed, and the measurement data was expressed as mean ± standard deviation (x ± s). Measurement data were analyzed by independent-sample t test, or Wilcoxon test or One-way ANOVA. Comparison of survival curves was used Log-rank (Mantel-Cox) test. Correlation analysis was used spearman correlation analysis. Drawing using Graphpad Prism 8.0. *P* ≤ 0.05 was considered statistically significant.

## Result

### Patient characteristics

In forty-six glioma patients, the number of IDH mutated patients were 21 (45.7%), IDH wild-type were 25 (54.3%), TERT mutated were 29(63%), TERT wild-type were 17(37%), MGMT promotor methylation were 33(71.7%), MGMT promotor un-methylation were 13(28.3%) respectively. Clinical characteristics such as age, gender, WHO grades, tumor histology and location were in Table 1, and more details were in supplementary Table 1.

Forty-four patients with WHO II-IV diffuse glioma were divided into four distinct subgroups based on IDH and TERT status. GBM was the most common in the group with mutation in TERT but not IDH (Group A) and the group with no detectable IDH or TERT mutations (Group B), accounting for 66.7 and 62.5% respectively. The group with mutations in both IDH and TERT (Group C) mainly consisted of oligodendroglioma (OL) or anaplastic oligodendrogliom (AO) (85.8%). The group with mutation in IDH but not TERT (Group D) mostly consisted of DA (71.4%). (Supplementary Table 2).

Niniteen GBM patients were divided into distinct subgroups based on MGMT and TERT status. The group with MGMT methylation but TERT wild-type included four patients (Group A). The group with MGMT methylation and TERT mutation included seven patients (Group B). The group with MGMT un-methylation and TERT wild-type included three patients (Group C). The group with MGMT un-methylation and TERT mutation included five patients (Group D). (Supplementary Table 3).

### Relationship between pathological grade and perfusion parameters

**Fig. 1:**
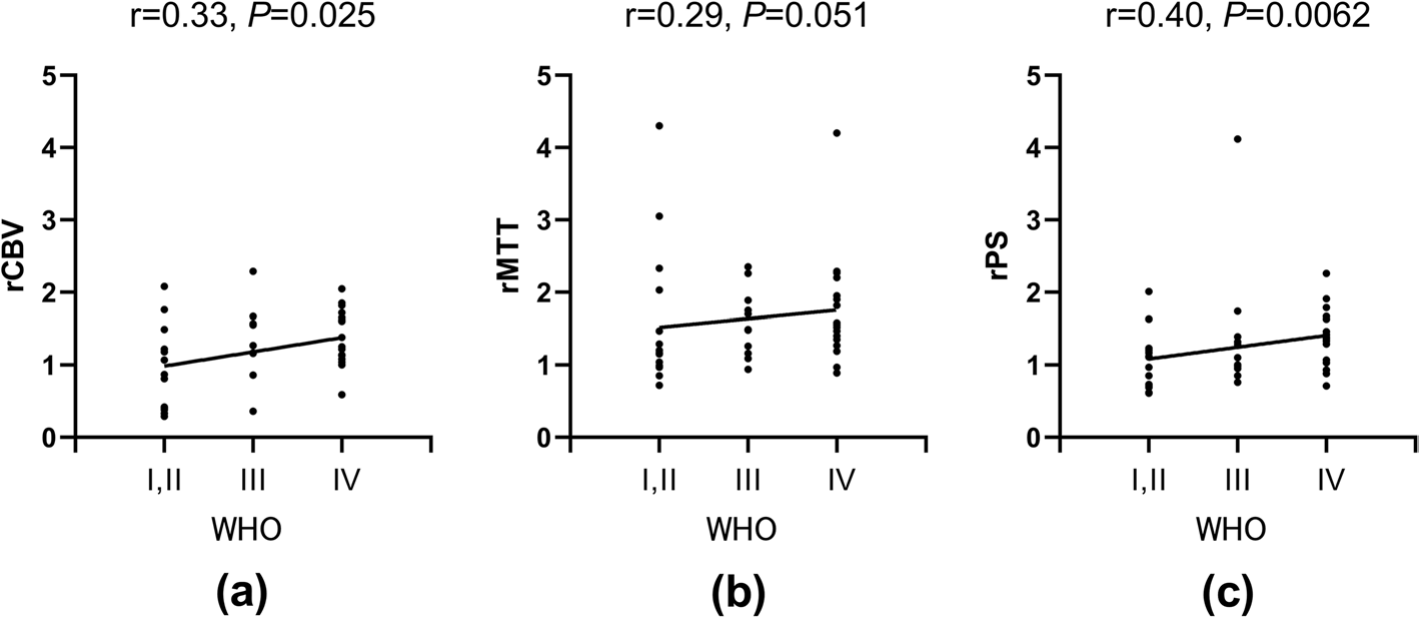
The correlation between WHO grades and perfusion parameters

In this study, there was only one patient with grade I glioma. So, the patients with grade I and grade II glioma were combined with analysis. Glioma grades was positively correlated with rCBV, rMTT and rPS in perfusion CT parameters (*P* < 0.05). (Table 2) With the increase of glioma grades, rCBV, rMTT and rPS showed an increasing trend. (Fig. 1).

**Table 2:** The correlation between tumor perfusion and WHO grade

Perfusion parameters	WHO	
	***r***	***p***
rCBV	0.331	0.025^*^
rCBF	0.054	0.722
rMTT	0.289	0.051^*^
rTTP	0.211	0.160
rPS	0.398	0.006^*^

* *P*<0.05

### Comparisons of perfusion parameters between different tumor biology markers

**Fig. 2:**
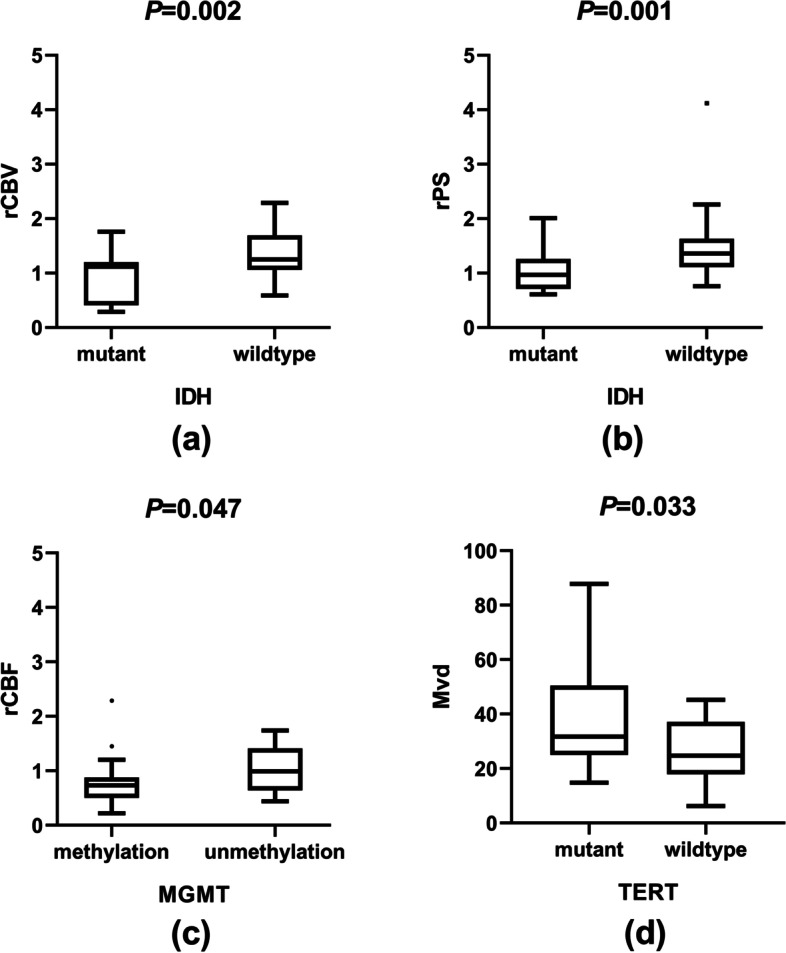
The comparisons of tumor biology markers and perfusion parameters

The group of IDH mutated and IDH wild-type show differences in rCBV and rPS on perfusion CT. The rCBV and rPS of IDH mutated group were lower than IDH wild-type group. (Table 3) The rCBF of MGMT methylation group was lower than un-methylation group. (Table 4) The MVD of TERT wild-type group was lower than TERT mutated group. (Table 5) The differences above were statistically significant (*P* < 0.05). (Fig. 2).

**Table 3:** The differences of perfusion parameters between IDH-wild-type and IDH-mutant

Perfusion parameters	IDH-mutant(***n*** = 21) (M ± SD)	IDH-wildtype(***n*** = 25) (M ± SD)	***t /Z***	***P***
rCBV	0.96 ± 0.45	1.39 ± 0.43	−3.38	0.002^*^
rCBF	0.8 ± 0.37	1.02 ± 0.93	0.45	0.651
rMTT	1.45 ± 0.53	1.81 ± 0.89	1.36	0.175
rTTP	1.08 ± 0.1	1.07 ± 0.1	0.03	0.974
rPS	1.02 ± 0.37	1.45 ± 0.65	3.25	0.001^*^
MVD	30.36 ± 14.75	35.68 ± 17.81	1.35	0.178

* *P*<0.05

**Table 4:** The differences of perfusion parameters between MGMT methylation and MGMT un-methylation

Perfusion parameters	Methylation(***n*** = 33) (M ± SD)	Unmythylation(***n*** = 13) (M ± SD)	***t/ Z***	***P***
rCBV	1.13 ± 0.48	1.36 ± 0.47	−1.15	0.142
rCBF	0.77 ± 0.38	1.29 ± 1.19	1.99	0.047^*^
rMTT	1.68 ± 0.72	1.56 ± 0.89	−1.04	0.300
rTTP	1.08 ± 0.11	1.06 ± 0.08	−0.79	0.428
rPS	1.26 ± 0.66	1.25 ± 0.32	0.48	0.634
MVD	33.41 ± 17.33	33.09 ± 15.15	− 0.03	0.980

* *P*<0.05

**Table 5:** The differences of perfusion parameters between TERT-mutant and TERT-wild-type

Perfusion parameters	TERT-mutant(***n*** = 29)(M ± SD)	TERT-wildtype(***n*** = 17)(M ± SD)	***t/ Z***	***P***
rCBV	1.26 ± 0.48	1.09 ± 0.49	1.16	0.251
rCBF	0.96 ± 0.83	0.84 ± 0.53	−0.876	0.381
rMTT	1.62 ± 0.68	1.69 ± 0.90	−0.292	0.772
rTTP	1.08 ± 0.08	1.06 ± 0.13	0.784	0.437
rPS	1.29 ± 0.67	1.20 ± 0.40	−0.148	0.882
MVD	37.40 ± 17.90	26.61 ± 11.69	2.209	0.033^*^

* *P*<0.05

### Comparisons of perfusion parameters, FPS and OS in the four molecular groups divided by the combined IDH/TERT classification in WHO II-IV diffuse glioma

**Fig. 3:**
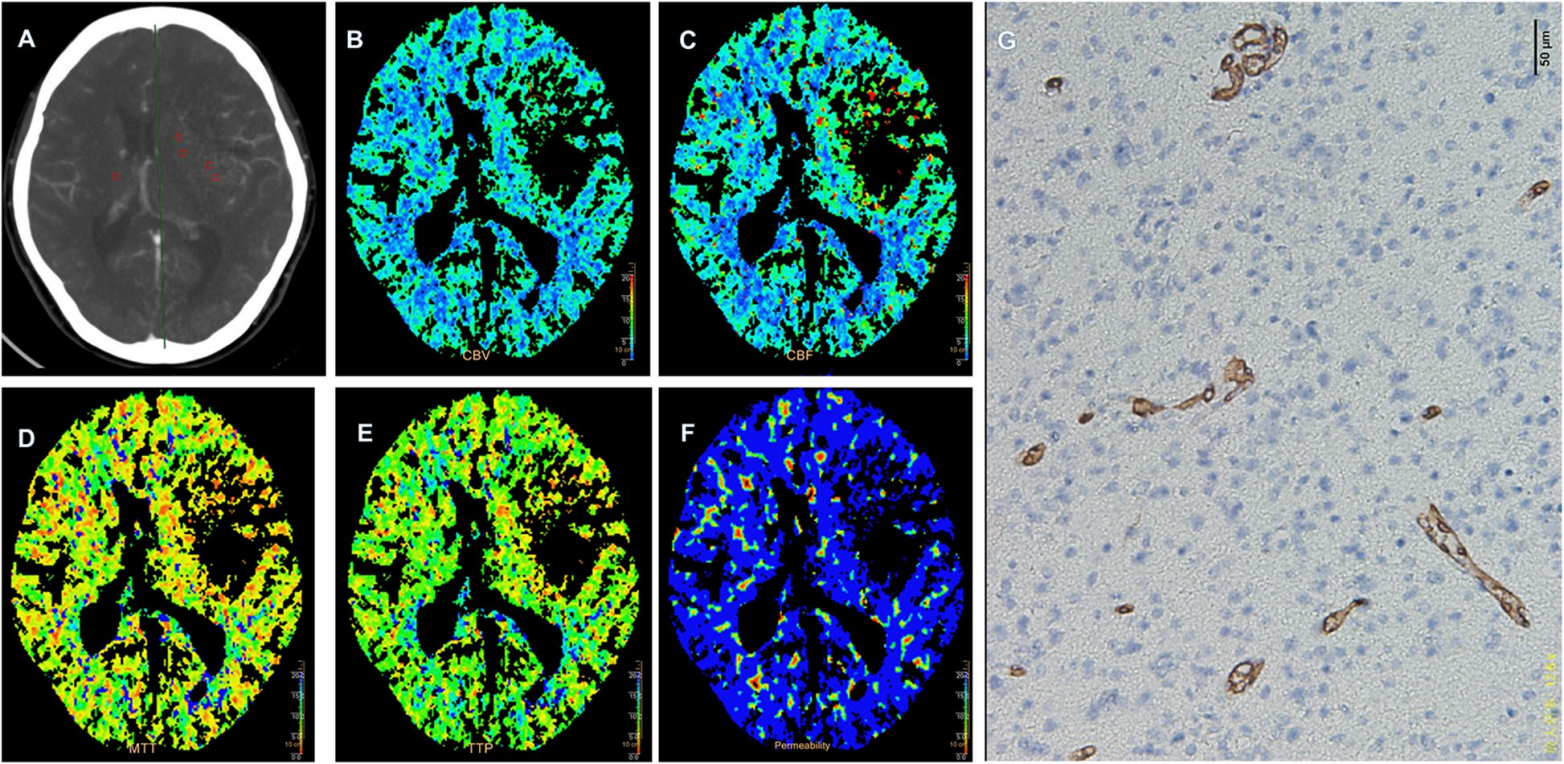
41-year-old woman with Left frontotemporal DA, WHO grade II, IDH-mut, TERT-wt, MGMT-un-met. **A** Transverse CT image and ROIs in red points. **B** PCT CBV map. **C** PCT CBF map. **D** MTT map. **E** TTP map. **F** PS map. **E** MVD map

**Fig. 4:**
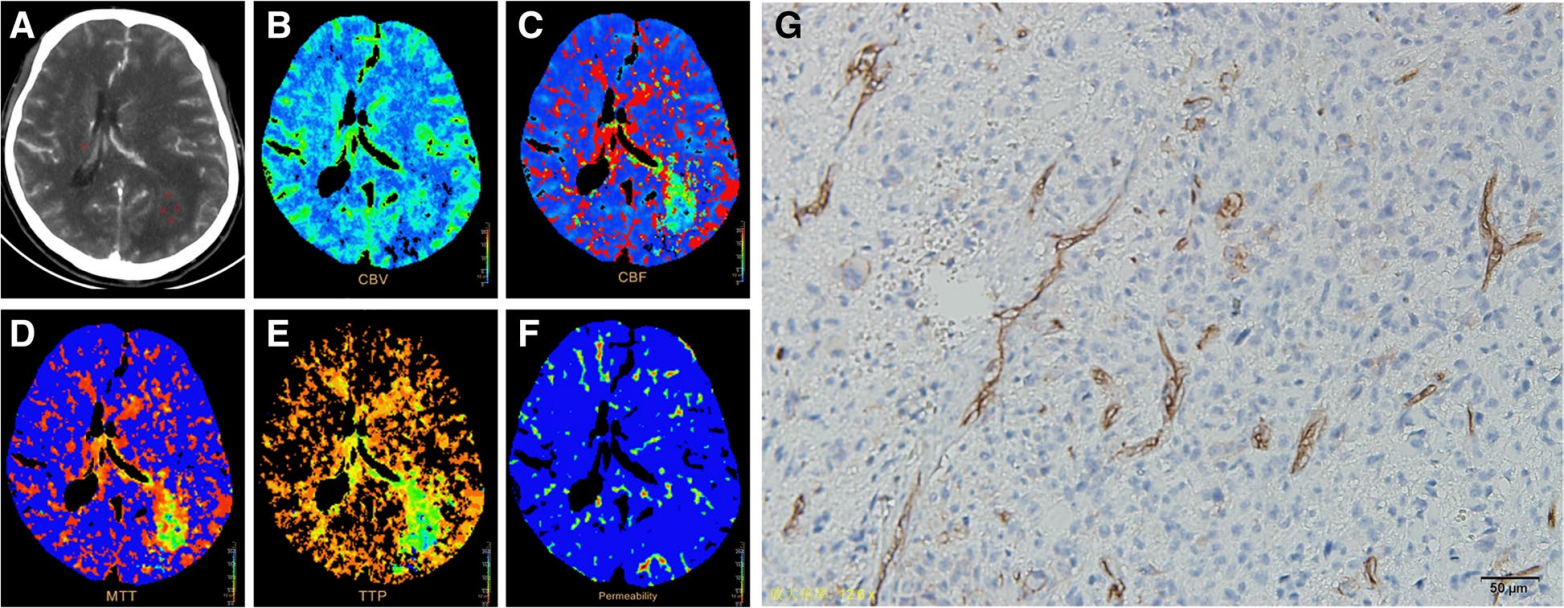
45-year-old man with Left frontal-parietal-occipital GBM, WHO grade IV, IDH-wt, TERT-mut, MGMT-met. **A** Transverse CT image and ROIs in red points. **B** PCT CBV map. **C** PCT CBF map. **D** MTT map. **E** TTP map. **F** PS map. **E** MVD map

**Fig. 5:**
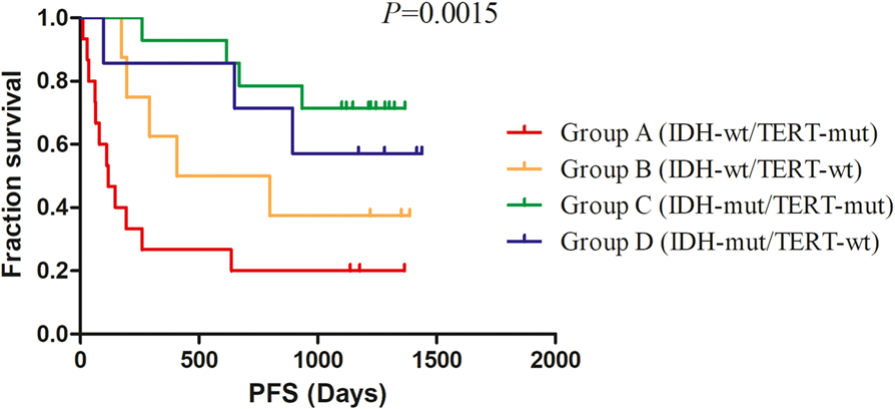
Comparisons of PFS in the four molecular groups divided by the combined IDH/TERT status in WHO II-IV diffuse glioma

**Fig. 6:**
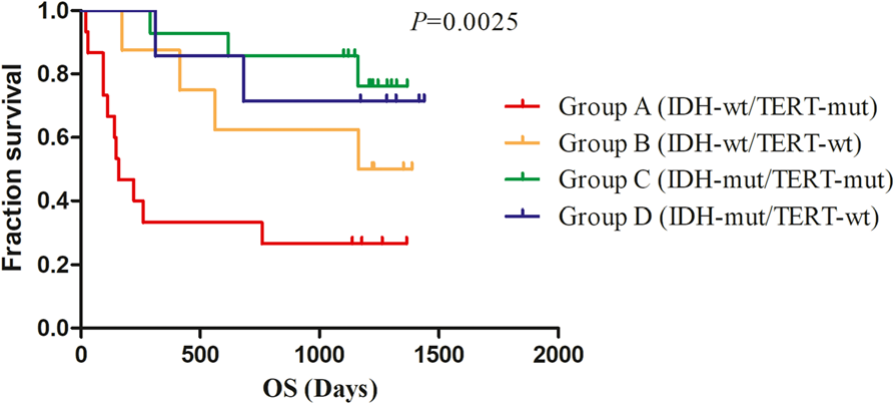
Comparisons of OS in the four molecular groups divided by the combined IDH/TERT status in WHO II-IV diffuse glioma

The results showed that the mean values of rCBV were statistically significant difference in the four molecular groups divided by the combined IDH/TERT classification (*P* < 0.05). PS, however, had a value close to statistical significances (*P* = 0.057). (Table 6, Figs. 3 and 4) Moreover, the results showed that the FPS and OS were statistically significant difference in the four molecular groups (*P* < 0.05). (Fig. 5 and 6) IDHwt/TERTmut group had the highest rCBV and the worst FPS and OS, and IDHmut/TERTmut group had the best FPS and OS. However, IDHmut/TERTwt group had the lower rCBV and higher rPS compared with IDHmut/TERTmut group.

**Table 6:** Comparisons of perfusion parameters in the four molecular groups divided by the combined IDH/TERT status in WHO II-IV glioma

Perfusion parameters	Group A (***n*** = 15) (M ± SD)	Group B (***n*** = 8) (M ± SD)	Group C (***n*** = 14) (M ± SD)	Group D (***n*** = 7) (M ± SD)	***F***	***P***
rCBV	1.45 ± 0.42	1.38 ± 0.46	1.05 ± 0.49	0.77 ± 0.40	4.962	0.005^*^
rCBF	1.14 ± 1.11	0.93 ± 0.60	0.77 ± 0.29	0.85 ± 0.52	0.603	0.617
rMTT	1.66 ± 0.83	1.82 ± 0.61	1.58 ± 0.51	1.20 ± 0.52	1.188	0.326
rTTP	1.08 ± 0.08	1.09 ± 0.09	1.09 ± 0.08	1.07 ± 0.14	0.081	0.970
rPS	1.56 ± 0.80	1.28 ± 0.31	1.01 ± 0.31	1.04 ± 0.49	2.729	0.057
MVD	39.96 ± 19.44	30.23 ± 14.81	31.29 ± 12.11	22.80 ± 7.86	2.229	0.101

Group A IDH wild-type-TERT mutated, Group B IDH wild-type-TERT wild-type, Group C IDH mutated-TERT mutated, Group D IDH mutated-TERT wild-type, * *P*<0.05

### Comparisons of perfusion parameters, FPS and OS in the four molecular groups divided by the combined MGMT/TERT classification in GBM

There were no statistically significant differences of the perfusion parameters in the four molecular groups divided by the combined MGMT/TERT classification in GBM. (Supplementary Table 3) But the results showed that the FPS and OS were statistically significant difference (*P* < 0.05). (Supplementary Fig. 1–2) The group with MGMT un-methylation and TERT mutation (Group D) had the worst FPS and OS.

## Discussion

This study was, to our knowledge, the first to analysis perfusion parameters evaluated by combining IDH mutation status and MGMT methylation status and TERT mutation status in glioma gene detection results. Previous studies have found that there is a significant difference in CBV, PS between LGGs and HGGs [20–22]. PCT parameters can relatively well distinguish patients with different histopathological grades of glioma. This was consistent with the increase in glioma malignancy with the increase in tumor grades. However, histopathological grading of the tumor had considerable limitations on distinguishing the malignancy of glioma. With the discovery and development of the genotyping methods of glioma pathological results, it had been proved that the genotyping results of glioma can more accurately judge the malignant degree of tumors than the results of histopathological grading [23, 24]. In this study, PCT related parameters were used to investigate the genotyping of pathological results based on the latest standards [16], hoping to provide more accurate biological markers for the diagnosis and treatment of glioma.

According to the literature, perfusion CT had several advantages compared with MRI biomarkers [13, 25, 26]: a. PCT was a widely available and cost effective neuroimaging method which was easy to perform on most new CT units. B. It required short scanning times, so it could be conducted without sedation, which was very important to the case of patients with severe symptoms, who were often uncooperative. C. The attenuation values and the contrast concentration of PCT were a more linear relationship and delivered a “superior quantitative accuracy” by providing absolute quantitative values of the perfusion parameters. D. With only one acquisition, PCT provides access to the usual parameters (CBV, CBF, MTT) as well as the permeability data.

According to our research, IDH wild-type tumors had higher rCBV, which may be related to the signaling pathways about a distinct transcriptome signature induced by upregulation of tumor cell hypoxia, and angiogenesis [21]. The IDH gene may promote signals related to tumor cell hypoxia, blood vessels and angiogenesis, accelerating the microangiogenesis of tumor cells. These signaling pathways are prerequisites for aggressive tumor behavior [27]. It may be related to the promotion of microvascular proliferation of tumor cells. Compared with IDH mutant gliomas, gliomas carrying the IDH wild-type gene are more aggressive.

The MGMT unmethylation glioma patients have higher rCBF in our study, which means the MGMT unmethylation glioma patients have more blood supply and faster blood flow in the tumor area. Previous findings showed that MGMT regulates angiogenesis in tumor cells by changing the levels of different vascular endothelial growth factor receptors [6]. Ahn et al. [28] had found that K^trans^ of perfusion MRI is associated with MGMT methylation status in glioblastoma, which indicating that MGMT methylation may be involved in glioma-associated angiogenesis characterized by high endothelial permeability vasculatures. Our results are consistent with previous results on the effect of MGMT on the prognosis of patients with glioma. Whether the prognosis of glioma patients with MGMT methylation is related not only to temozolomide sensitivity, but also to the decrease of vascular endothelial permeability needs further study. Gliomas with the TERT mutation had higher MVD compared with TERT wild-type in our study. This is consistent with the latest study in which TERT promoter mutations improved gene transcription and resulted in increased TERT mRNA levels, which leads to a corresponding poor prognosis in patients [8].

Due to tumor was regulated by different genes at the same time, our study firstly attempted to group a number of glioma genes with different tumor grades, and explore the difference of perfusion results, PFS, and OS between different groups. Previous study [20] had showed that IDHwt/TERTmut group has the worst FPS and OS, and IDHmut/TERTmut group had the best FPS and OS. Our results were consistent with it. Moreover, in our study, IDHwt/TERTmut group had the highest rCBV, and IDHmut/TERTwt group had the lower rCBV and higher rPS compared with IDHmut/TERTmut group, which may imply the IDH/TERT status and prognosis could be predicted by rCBV. In previous study [20, 29], CBV and PS reflected vascular density and vascular permeability, respectively, and therefore the two components of tumor neovascularity, which had an additive and not an exclusive effect on the prognosis of glioma. We speculated that the better prognosis of IDHmut/TERTmut group may be related to vascular permeability and other effects, which need to be further studied in a larger series of patients. There were no significant differences in the four molecular groups by grouping based on the combined MGMT/TERT status in GBM. However, the PFS and OS showed statistically significant differences. The group with MGMT un-methylation and TERT mutation had the worst FPS and OS. Due to the samples were relatively small, this needed to be further studied in a larger series of patients with GBM.

### Clinical implications

This study was, to our knowledge, the first to analysis perfusion parameters were evaluated by combining IDH mutation status and TERT mutation status in glioma gene detection results. The findings of our study were: 1) with the increase of glioma grade, rCBV, rMTT and rPS showed an increasing trend. 2) the rCBV and rPS of IDH mutated group were lower than IDH wild-type group. 3) the rCBF of MGMT methylation group were lower than un- methylation group. 4) In WHO II-IV diffuse gliomas, the rCBV was closely related to IDH combined with TERT status, and the higher rCBV could indicate the worse prognosis.

### Study limitations

There were some limitations in our study. Firstly, the samples of this study were relatively small because relatively few patients had perfusion CT scans, and which could result in a possible bias. Secondly, our study may have sampling bias becauses the the specimen might not have corresponded to the intended area of PCT map. Future studies should replicate this study in larger samples, and combined with the use of other advanced neuroimaging techniques.

## Conclusions

To summarize, perfusion parameters of glioma maybe related to the degree of tumor malignancy and the status of IDH, MGMT and TERT. The rCBV maybe an important predictive imaging marker of the combined IDH/TERT status and prognosis in WHO II-IV diffuse glioma.

## Supplementary Information

**Additional file 1: Supplementary Table 1.** Patient background and molecular status. **Supplementary Table 2.** Patient background of four molecular groups divided by the combined IDH/TERT status in WHO II-IV diffuse glioma. **Supplementary Table 3. **Comparisons of perfusion parameters in the four molecular groups divided by the combined MGMT/TERT status in GBM.

**Additional file 2: Supplementary Fig. 1.** Comparisons of PFS in the four molecular groups divided by the combined MGMT/TERT status in GBM.

**Additional file 3: Supplementary Fig. 2.** Comparisons of OS in the four molecular groups divided by the combined MGMT/TERT status in GBM.

## Data Availability

The datasets used and analyzed during the current study available from the corresponding author on reasonable request.

## Abbreviations

AA: Anaplastic astrocytoma
AO: Anaplastic oligodendrogliom
CBF: Cerebral blood flow
CBV: Cerebral blood volume
clMPACT-NOW: Consortium to Inform Molecular and Practical Approaches to CNS Tumor Taxonomy
DA: Diffuse astrocytoma
GBM: Glioblastoma
IDH: Isocitrate dehydrogenase
KPS: Karnofsky performance status
MGMT: O6-methylguanine-DNA-methyltransferase
MRI: Magnetic resonance imaging
MTT: Mean transition time
MVD: Microvessel density
OL: Oligodendroglioma
OS: Overall survival
PCT: Perfusion computed tomography
PFS: Progression free survival
PS: Permeability surface
ROIs: Regions of interests
WHO: World health organization

## References

[1] Stoecklein VM, Stoecklein S, Galiè F, Ren J, Schmutzer M, Unterraine M (2020). Resting-state fMRI detects alterations in whole brain connectivity related to tumor biology in glioma patients. Neuro Oncol.

[2] Houillier C, Wang X, Kaloshi G, Mokhtari K, Guillevin R, Laffaire J (2010). IDH1 or IDH2 mutations predict longer survival and response to temozolomide in low-grade gliomas. Neurology.

[3] Zhang J, Yang J-H, Quan J, Kang X, Wang H-J, Dai P-G (2016). Identification of MGMT promoter methylation sites correlating with gene expression and IDH1 mutation in gliomas. Tumour Biol.

[4] Eckel-Passow JE, Lachance DH, Molinaro AM, Walsh KM, Decker PA, Sicotte H (2015). Glioma Groups Based on 1p/19q, IDH, and TERT Promoter Mutations in Tumors. New Engl J Med.

[5] Baylin SB, Ohm JE (2006). Epigenetic gene silencing in cancer - a mechanism for early oncogenic pathway addiction?. Nat Rev Cancer.

[6] Chahal M, Xu Y, Lesniak D, Graham K, Famulski K, Christensen JG (2010). MGMT modulates glioblastoma angiogenesis and response to the tyrosine kinase inhibitor sunitinib. Neuro-Oncology.

[7] Simon M, Hosen I, Gousias K, Rachakonda S, Heidenreich B, Gessi M (2015). TERT promoter mutations: a novel independent prognostic factor in primary glioblastomas. Neuro-Oncology.

[8] Powter B, Jeffreys SA, Sareen H, Cooper A, Brungs D, Po J (2021). Human TERT promoter mutations as a prognostic biomarker in glioma. J Cancer Res Clin Oncol.

[9] Hardee ME, Zagzag D (2012). Mechanisms of glioma-associated neovascularization. Am J Pathol.

[10] Jain R, Griffith B, Alotaibi F, Zagzag D, Fine H, Golfinos J (2015). Glioma angiogenesis and perfusion imaging: understanding the relationship between tumor blood volume and leakiness with increasing glioma grade. AJNR Am J Neuroradiol.

[11] Ellika SK, Jain R, Patel SC, Scarpace L, Schultz LR, Rock JP, Mikkelsen T (2007). Role of perfusion CT in glioma grading and comparison with conventional MR imaging features. AJNR Am J Neuroradiol.

[12] Ahmad N, Shaukat A, Rehan A, Rashid S (2016). Diagnostic accuracy of perfusion computed tomography in cerebral glioma grading. J Coll Physicians Surg Pak.

[13] Karegowda LH, Kadavigere R, Shenoy PM, Paruthikunnan SM (2017). Efficacy of Perfusion Computed Tomography (PCT) in Differentiating High-Grade Gliomas from Low Grade Gliomas, Lymphomas, Metastases and Abscess. J Clin Diagn Res.

[14] Kaichi Y, Tatsugami F, Nakamura Y, Baba Y, Iida M, Higaki T (2018). Improved differentiation between high- and low-grade gliomas by combining dual-energy CT analysis and perfusion CT. Medicine (Baltimore).

[15] Onishi S, Kajiwara Y, Takayasu T, Kolakshyapati M, Ishifuro M, Amatya VJ (2018). Perfusion Computed Tomography Parameters Are Useful for Differentiating Glioblastoma, Lymphoma, and Metastasis. World Neurosurg.

[16] Brat DJ, Aldape K, Colman H, Holland EC, Louis DN, Jenkins RB (2018). cIMPACT-NOW update 3: recommended diagnostic criteria for “Diffuse astrocytic glioma, IDH-wildtype, with molecular features of glioblastoma, WHO grade IV”. Acta Neuropathol.

[17] Yang P, Cai J, Yan W, Zhang W, Wang Y, Chen B (2016). Classification based on mutations of TERT promoter and IDH characterizes subtypes in grade II/III gliomas. Neuro-Oncology.

[18] Arita H, Yamasaki K, Matsushita Y, Nakamura T, Shimokawa A, Takami H (2016). A combination of TERT promoter mutation and MGMT methylation status predicts clinically relevant subgroups of newly diagnosed glioblastomas. Acta Neuropathol Commun.

[19] Narang J, Jain R, Scarpace L, Saksena S, Schultz LR, Rock JP (2011). Tumor vascular leakiness and blood volume estimates in oligodendrogliomas using perfusion CT: an analysis of perfusion parameters helping further characterize genetic subtypes as well as differentiate from astroglial tumors. J Neuro-Oncol.

[20] Shankar JJS, Woulfe J, Da Silva V, Nguyen TB (2013). Evaluation of perfusion CT in grading and prognostication of high-grade gliomas at diagnosis: a pilot study. AJR Am J Roentgenol.

[21] Kickingereder P, Sahm F, Radbruch A, Wick W, Heiland S, von Deimling A (2015). IDH mutation status is associated with a distinct hypoxia/angiogenesis transcriptome signature which is non-invasively predictable with rCBV imaging in human glioma. Sci Rep.

[22] Fainardi E, Di Biase F, Borrelli M, Saletti A, Cavallo M, Sarubbo S (2010). Potential role of CT perfusion parameters in the identification of solitary intra-axial brain tumor grading. Acta Neurochir Suppl.

[23] Chai R, Li G, Liu Y, Zhang K, Zheng Z, Wu F (2021). Predictive value of MGMT promoter methylation on the survival of TMZ treated IDH-mutant glioblastoma. Cancer Biol Med.

[24] Jain R, Ellika SK, Scarpace L, Schultz LR, Rock JP, Gutierrez J (2008). Quantitative estimation of permeability surface-area product in astroglial brain tumors using perfusion CT and correlation with histopathologic grade. AJNR Am J Neuroradiol.

[25] Zimny A, Leszek J, Kiejna A, Sasiadek M (2007). Analysis of correlation between the degree of cognitive impairment and the results of perfusion CT in patients with dementia. Med Sci Monit.

[26] Grand S, Tahon F, Attye A, Lefournier V, Le Bas J-F, Krainik A (2013). Perfusion imaging in brain disease. Diagn Interv Imaging.

[27] Chesnelong C, Chaumeil MM, Blough MD, Al-Najjar M, Stechishin OD, Chan JA (2014). Lactate dehydrogenase a silencing in IDH mutant gliomas. Neuro-Oncology.

[28] Ahn SS, Shin N-Y, Chang JH, Kim SH, Kim EH, Kim DW (2014). Prediction of methylguanine methyltransferase promoter methylation in glioblastoma using dynamic contrast-enhanced magnetic resonance and diffusion tensor imaging. J Neurosurg.

[29] Jain R, Gutierrez J, Narang J, Scarpace L, Schultz LR, Lemke N (2011). In vivo correlation of tumor blood volume and permeability with histologic and molecular angiogenic markers in gliomas. AJNR Am J Neuroradiol.

