# Supplementary material for: Perfusion CT detects alterations in local cerebral flow of glioma related to IDH, MGMT and TERT status
Source: BMC Neurol. 2021 Nov 24;21:460. doi: 10.1186/s12883-021-02490-4 (PMC8611974; doi:10.1186/s12883-021-02490-4)
Supplement: Supplementary file 1 — Additional file 1: Supplementary Table 1. Patient background and molecular status. Supplementary Table 2. Patient background of four molecular groups divided by the combined IDH/TERT status in WHO II-IV diffuse glioma. Supplementary Table 3. Comparisons of perfusion parameters in the four molecular groups divided by the combined MGMT/TERT status in GBM. [file 12883_2021_2490_MOESM1_ESM.docx]

**Supplementary table 1**

| **No.** | **Gender** | **Age (year)** | **Location** | **WHO grade** | **Histological subtype** | **MGMT** | **IDH** | **TERT** | **BRAF** | **Total 1p/19q loss** | **Adjuvant**  **therapy** | **PFS**  **(day)** | **OS (day)** |
| --- | --- | --- | --- | --- | --- | --- | --- | --- | --- | --- | --- | --- | --- |
| 1 | M | 24 | Cerebellar vermis | 1 | Pilocytic Astrocytoma | Un-met | Wt | Wt | Mut | No | None | None | None |
| 2 | M | 36 | Left frontotemporal lobe and insula | 2 | DA | Met | Mut | Wt | Wt | No | None | None | None |
| 3 | F | 47 | Right frontotemporal lobe | 2 | OL | Met | Mut | Mut | Wt | Yes | Chemo | No recurrence | Survival |
| 4 | F | 41 | Left frontotemporal lobe | 2 | DA | Un-met | Mut | Wt | Wt | No | None | No recurrence | Survival |
| 5 | F | 23 | Left frontal lobe | 2 | DA | Met | Mut | Wt | Wt | No | Chemo | 894 | Survival |
| 6 | F | 27 | Right frontal lobe | 2 | DA | Met | Mut | Wt | Wt | No | Chemo | No recurrence | Survival |
| 7 | F | 49 | Right occipital-parietal lobe | 2 | DA | Met | Mut | Wt | Wt | No | Chemo | No recurrence | Survival |
| 8 | F | 51 | Right temporal-occipital lobe | 2 | OL | Met | Mut | Mut | Wt | Yes | None | 933 | 1160 |
| 9 | F | 54 | Left frontal lobe | 2 | OL | Met | Mut | Mut | Wt | Yes | Chemo | No recurrence | Survival |
| 10 | M | 55 | Left frontal lobe | 2 | OL | Met | Mut | Mut | Wt | Yes | None | 261 | 290 |
| 11 | F | 72 | Left frontal lobe | 2 | DA | Met | Wt | Mut | Wt | No | Chemo | No recurrence | Survival |
| 12 | F | 26 | Left temporal lobe | 2 | Pleomorphic Xantho Astrocytoma | Met | Wt | Wt | Wt | No | Chemo | No recurrence | Survival |
| 13 | F | 50 | Right frontal lobe | 2 | OL | Met | Mut | Mut | Wt | Yes | Chemo | No recurrence | Survival |
| 14 | F | 83 | Left cerebellum | 2 | DA | Met | Wt | Wt | Wt | No | Chemo | No recurrence | Survival |
| 15 | F | 15 | Left frontal lobe and Basal ganglia | 2 | DA | Met | Wt | Wt | Wt | No | Chemo | 406 | Survival |
| 16 | M | 38 | Left frontal lobe | 2 | OL | Met | Mut | Mut | Wt | Yes | Chemo | No recurrence | Survival |
| 17 | M | 60 | Right frontal-parietal lobe | 3 | AO | Met | Mut | Mut | Wt | Yes | None | None | None |
| 18 | F | 43 | Right temporal-parietal-occipital lobe | 3 | AA | Met | Wt | Mut | Wt | No | None | None | 147 |
| 19 | F | 42 | Right temporal lobe | 3 | AA | Un-met | Wt | Wt | Mut | No | Chemo | No recurrence | Survival |
| 20 | M | 50 | Left frontal lobe | 3 | AO | Met | Mut | Mut | Wt | Yes | Chemo | None | 616 |
| 21 | F | 47 | Left frontal-parietal lobe | 3 | AO | Met | Wt | Mut | Wt | Yes | Chemo | No recurrence | Survival |
| 22 | M | 40 | Right frontal lobe | 3 | AO | Met | Mut | Mut | Wt | Yes | Chemoradiotherapy | No recurrence | Survival |
| 23 | F | 55 | Left frontal lobe | 3 | AO | Met | Mut | Mut | Wt | Yes | Chemo | No recurrence | Survival |
| 24 | M | 39 | Left temporal-parietal lobe | 3 | AA | Un-met | Wt | Mut | Wt | No | None | 117 | Survival |
| 25 | F | 59 | Bilateral corpus callosum and frontal lobe | 3 | AA | Un-met | Wt | Mut | Wt | No | Chemo | None | 260 |
| 26 | F | 40 | Left parietal lobe | 3 | AO | Met | Mut | Mut | Wt | Yes | Chemoradiotherapy | No recurrence | Survival |
| 27 | F | 45 | Right parietal lobe | 3 | AO | Met | Mut | Mut | Wt | Yes | Chemoradiotherapy | No recurrence | Survival |
| 28 | M | 45 | Left frontal-parietal-occipital lobe | 4 | GBM | Met | Wt | Mut | Wt | No | Chemo | No recurrence | Survival |
| 29 | M | 65 | Right parietal-occipital lobe | 4 | GBM | Met | Wt | Wt | Wt | No | None | 797 | 1162 |
| 30 | M | 49 | Left frontotemporal lobe | 4 | GBM | Met | Mut | Mut | Wt | No | None | None | None |
| 31 | F | 46 | Left frontotemporal-parietal lobe | 4 | GBM | Un-met | Wt | Wt | Mut | No | Chemoradiotherapy | No recurrence | Survival |
| 32 | M | 45 | Left frontal-parietal lobe | 4 | GBM | Un-met | Wt | Mut | Wt | No | Chemo | 65 | 94 |
| 33 | M | 67 | Right frontal lobe | 4 | GBM | Met | Wt | Mut | Wt | No | Chemo | 63 | 94 |
| 34 | F | 50 | Right temporal-parietal-occipital lobe | 4 | GBM | Un-met | Wt | Mut | Wt | No | Chemo | 35 | 157 |
| 35 | F | 70 | Left temporal-occipital lobe | 4 | GBM | Met | Wt | Wt | Wt | No | Chemo | 292 | 414 |
| 36 | M | 50 | Left parietal-occipital lobe | 4 | GBM | Met | Mut | Wt | Wt | No | Chemo | 98 | 312 |
| 37 | M | 58 | Right frontal-parietal lobe | 4 | GBM | Met | Wt | Mut | Wt | No | Chemoradiotherapy | 636 | 758 |
| 38 | M | 51 | Right parietal lobe | 4 | GBM | Un-met | Wt | Mut | Wt | No | None | None | 29 |
| 39 | M | 30 | Right temporal lobe | 4 | GBM | Met | Wt | Wt | Wt | No | None | None | 173 |
| 40 | M | 51 | Left frontal lobe | 4 | GBM | Un-met | Mut | Wt | Wt | No | Chemoradiotherapy | 649 | 680 |
| 41 | F | 61 | Bilateral frontal lobe | 4 | GBM | Met | Wt | Mut | Wt | No | Chemo | None | 111 |
| 42 | M | 54 | Right frontotemporal-parietal lobe | 4 | GBM | Met | Mut | Mut | Wt | Yes | None | 669 | Survival |
| 43 | M | 45 | Left temporal-occipital lobe | 4 | GBM | Un-met | Wt | Mut | Mut | No | Chemoradiotherapy | 80 | 141 |
| 44 | M | 54 | Right temporal lobe | 4 | GBM | Un-met | Wt | Wt | Wt | No | None | 195 | 560 |
| 45 | M | 62 | Right corpus callosum | 4 | GBM | Met | Wt | Mut | Wt | No | Chemo | 193 | 222 |
| 46 | F | 62 | Left frontal lobe | 4 | GBM | Un-met | Wt | Mut | Wt | No | None | 10 | 30 |
| AA anaplastic astrocytoma, AO anaplastic oligodendrogliom, Chemo Chemotherapy, CRT chemoradiotherapy, DA diffuse astrocytoma, F fmale, GBM glioblastoma, M male, Met Met, Mut Mutated, None no information, OL oligodendroglioma, OS overall survival, PFS progression free survival, RT radiation therapy, Un-met Un-met, Wt Wt-type | | | | | | | | | | | | | |

**Supplementary table 2**

| **Characteristics** | **Group A (n=15)** | **Group B (n=8)** | **Group C (n=14)** | **Group D (n=7)** |
| --- | --- | --- | --- | --- |
| **Gender**(male/female) | 8/7 | 3/5 | 7/7 | 3/4 |
| **Age**(ys), (Mean ± SD) | 53.73±9.90 | 50.63±22.14 | 14.00±6.51 | 39.57±11.37 |
| **Tumor Histology** |  |  |  |  |
| **DA** | 1(6.7%) | 2(25%) | 0 | 5(71.4%) |
| **AA** | 2(13.3%) | 1(12.5%) | 0 | 0 |
| **AO** | 2(13.3%) | 0 | 6(42.9%) | 0 |
| **OL** | 0 | 0 | 6(42.9%) | 0 |
| **GBM** | 10(66.7%) | 5(62.5%) | 2(14.2%) | 2(28.6%) |
| Group A IDH wild-type-TERT mutated, Group B IDH wild-type-TERT wild-type, Group C IDH mutated-TERT mutated, Group D IDH mutated-TERT wild-type, AA anaplastic astrocytoma, AO anaplastic oligodendrogliom, DA diffuse astrocytoma, F fmale, GBM glioblastoma, M male, OL oligodendroglioma | | | | |

**Supplementary table 3**

| **Perfusion**  **parameters** | **GroupA (n=4)**  **(M±SD)** | **GroupB (n=7)**  **(M±SD)** | **GroupC (n=3)**  **(M±SD)** | **GroupD (n=5)**  **(M±SD)** | ***F*** | ***P*** |
| --- | --- | --- | --- | --- | --- | --- |
| rCBV | 1.31±0.34 | 1.45±0.35 | 0.99±0.35 | 1.44±0.40 | 1.287 | 0.315 |
| rCBF | 0.77±0.32 | 0.75±0.21 | 0.84±0.45 | 0.89±0.32 | 0.256 | 0.856 |
| rMTT | 1.82±0.36 | 1.73±0.47 | 1.55±0.65 | 1.92±1.30 | 0.152 | 0.927 |
| rTTP | 1.14±0.04 | 1.09±0.81 | 1.00±0.05 | 1.09±0.08 | 2.133 | 0.139 |
| rPS | 1.29±0.18 | 1.49±0.46 | 1.29±0.55 | 1.29±0.34 | 0.355 | 0.786 |
| MVD | 28.14±18.46 | 34.57±24.73 | 31.86±11.22 | 37.33±17.16 | 0.167 | 0.917 |
| Group A MGMT met-TERT wild-type, Group B MGMT met-TERT mutated, Group C MGMT un-met-TERT wild-type, Group D MGMT un-met-TERT mutated | | | | | | |
