# Supplementary figures and images for: Perfusion CT detects alterations in local cerebral flow of glioma related to IDH, MGMT and TERT status
Source: BMC Neurol. 2021 Nov 24;21:460. doi: 10.1186/s12883-021-02490-4 (PMC8611974; doi:10.1186/s12883-021-02490-4)

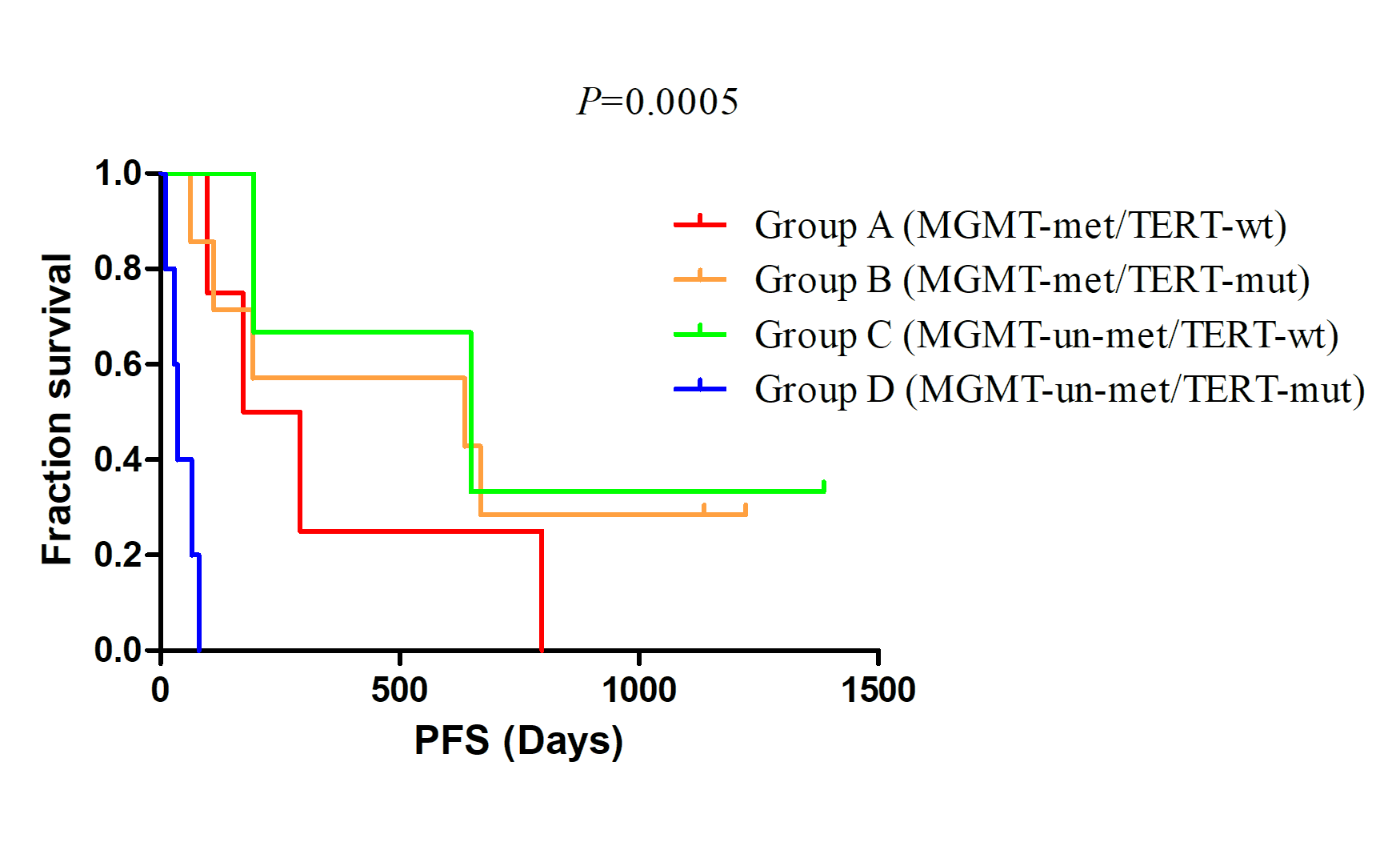

Supplement: Supplementary file 2 — Additional file 2: Supplementary Fig. 1. Comparisons of PFS in the four molecular groups divided by the combined MGMT/TERT status in GBM. [file 12883_2021_2490_MOESM2_ESM.tif]

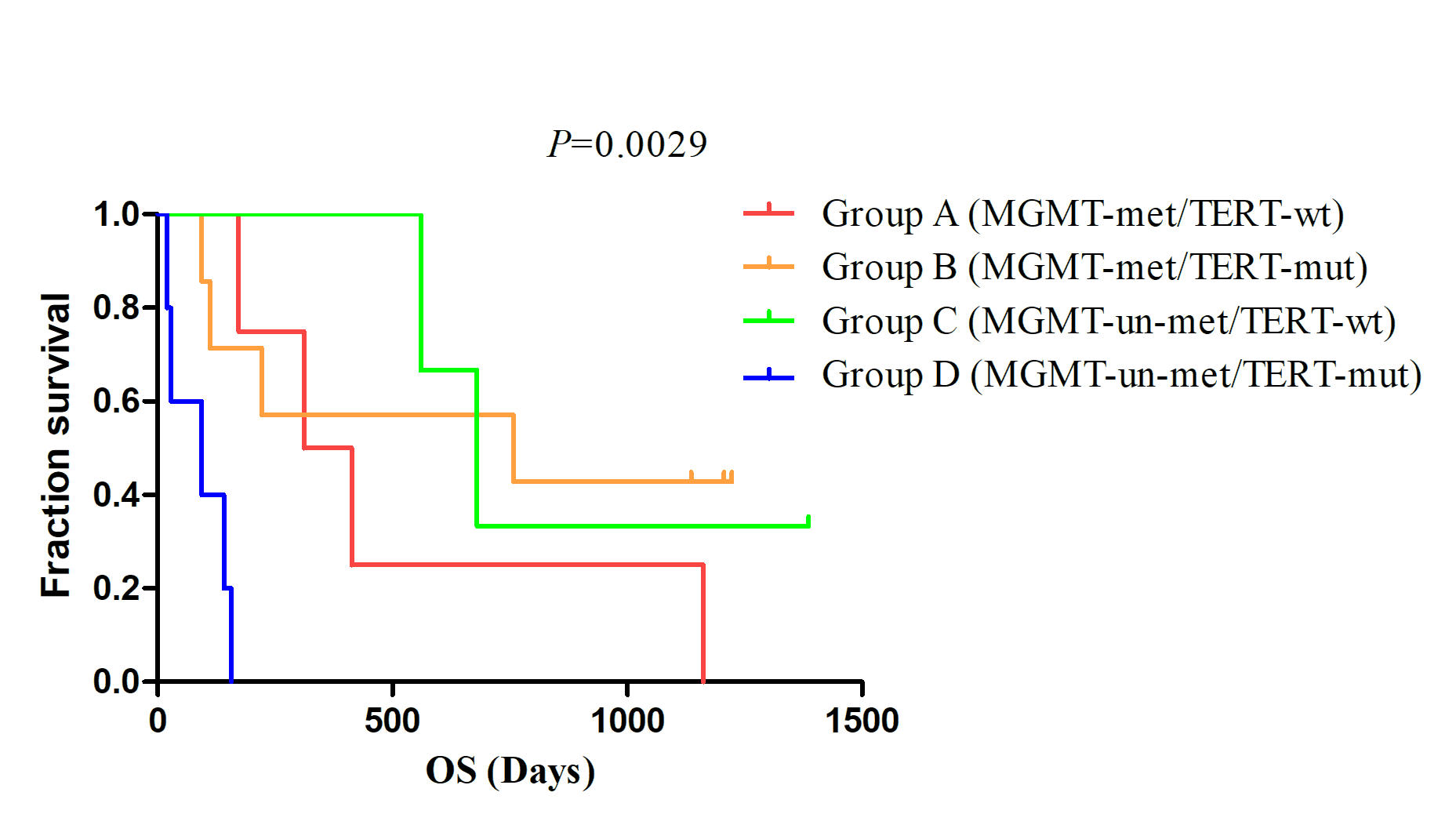

Supplement: Supplementary file 3 — Additional file 3: Supplementary Fig. 2. Comparisons of OS in the four molecular groups divided by the combined MGMT/TERT status in GBM. [file 12883_2021_2490_MOESM3_ESM.tif]
